# A study on the causes of operative failures after microwave ablation for primary hyperparathyroidism

**DOI:** 10.1007/s00330-021-07761-9

**Published:** 2021-03-02

**Authors:** Wei Ying, Zhao Zhen-long, Cao Xiao-jing, Peng Li-li, Li Yan, Yu Ming-an

**Affiliations:** Department of Interventional Medicine, China-Japan Friendship Hospital, No. 2 Ying-hua-yuan street, Chao-yang District, Beijing, 100029 China

**Keywords:** Microwave radiation, Primary hyperparathyroidism, Parathyroid Hormone, Recurrence

## Abstract

**Objective:**

To summarize the occurrence of operative failures after microwave ablation (MWA) in patients with primary hyperparathyroidism (pHPT), analyze the possible reasons, and explore strategies for preventing and managing these situations.

**Methods:**

This retrospective study reviewed 91 pHPT patients who underwent MWA from April 2015 to November 2019. A cure was defined as the reestablishment of normal calcium homeostasis lasting a minimum of 6 months. An operative failure was defined as a failure to normalize serum intact parathyroid hormone (iPTH) and/or calcium levels at 6 months or longer. Patients who encountered operative failures were compared with patients who were successfully cured.

**Results:**

Eighty-eight pHPT patients, consisting of 29 men and 59 women, were finally enrolled. The median follow-up duration was 15.9 months (IQR, 6.1–31.5 months). Seventy-eight patients (78/88, 88.6%) were cured. Ten (10/88, 11.4%) patients experienced operative failure, including 9 persistent pHPT (10.2%) and 1 (1.1%) recurrent pHPT. Small parathyroid nodules (maximum diameter < 0.6 cm) and incomplete ablation were the two key factors leading to operative failure. Of the 9 patients with a maximum nodule diameter less than 0.6 cm, 77.8% (7/9) of them encountered operative failure.

**Conclusion:**

Operative failure occurred in 11.4% of the pHPT patients who underwent MWA. The possibility of operative failure was increased when the maximum diameter of parathyroid nodule was less than 0.6 cm. Complete ablation could help avoid operative failure.

**Key Points:**

*• Failed to ablate the target lesion and incomplete ablation were the key factors attributed to operative failures.*

*• When the maximum diameter of the parathyroid nodules is less than 0.6 cm, the possibility of operative failure was higher.*

## Introduction

Primary hyperparathyroidism (pHPT) is a common endocrine disorder, with a prevalence of 0.1–0.4% [1]. It is generally characterized by hypercalcemia and elevated parathyroid hormone (PTH) levels [2]. Nearly 20% of pHPT patients are asymptomatic when discovered; however, the disease has the potential to become symptomatic. The common clinical manifestations of pHPT include bone loss, kidney stones, and neurocognitive impairment [1–4].

Surgical resection is recommended as the definitive curative treatment for pHPT [5–7]. In recent years, after more than a decade of clinical application, ultrasound-guided thermal ablation has been demonstrated to be effective in inactivating parathyroid nodules and normalizing serum PTH and calcium levels [8–12]. A recent prospective study showed that microwave ablation (MWA) and surgical resection provided comparable results in terms of cure rate in the treatment of pHPT [10].

Despite these high cure rates, approximately 11–18% of patients did not achieve reestablished normal serum PTH and/or calcium homeostasis after MWA [10, 11]. It was reported that the most common causes of initial operative failure after surgical resection include unrecognized gland hyperplasia, double adenomas, ectopic location of the hyperfunctioning parathyroid gland, or operations performed by inexperienced or low-volume parathyroid surgeons [13–15]. However, currently, operative failures after MWA have not been systematically summarized and analyzed. Therefore, the purpose of this study was to summarize the occurrence of operative failures after MWA for pHPT, analyze the possible reasons, and explore strategies for preventing and managing these situations.

## Materials and methods

### Study design

This retrospective study protocol was approved by the Human Ethics Review Committee of the China-Japan Friendship Hospital. Written informed consent was obtained from each patient before the ablation procedure; informed consent was waived for this retrospective investigation. Between April 2015 and November 2019, the data of 91 pHPT patients (30 male and 61 female) treated with MWA were reviewed.

The inclusion criteria were as follows: (1) patients with symptomatic pHPT; (2) asymptomatic pHPT patients with one of the following conditions—(a) serum calcium level higher than the normal range; (b) T-score < −2.5 at the lumbar spine, total hip, femoral neck, or distal one-third of the radius, significant reduction in bone mineral density, and/or increased risk of a fragility fracture; (c) age < 50 years; (3) patients with renal involvement—(a) creatinine clearance less than 60 ml/min, (b) kidney stone or nephrocalcinosis found by ultrasound (US), CT, or abdominal radiography; (4) pHPT patients who were not eligible for surgery or refused surgery; and (5) pHPT patients unwilling to comply with observation protocols.

**Fig. 1:**
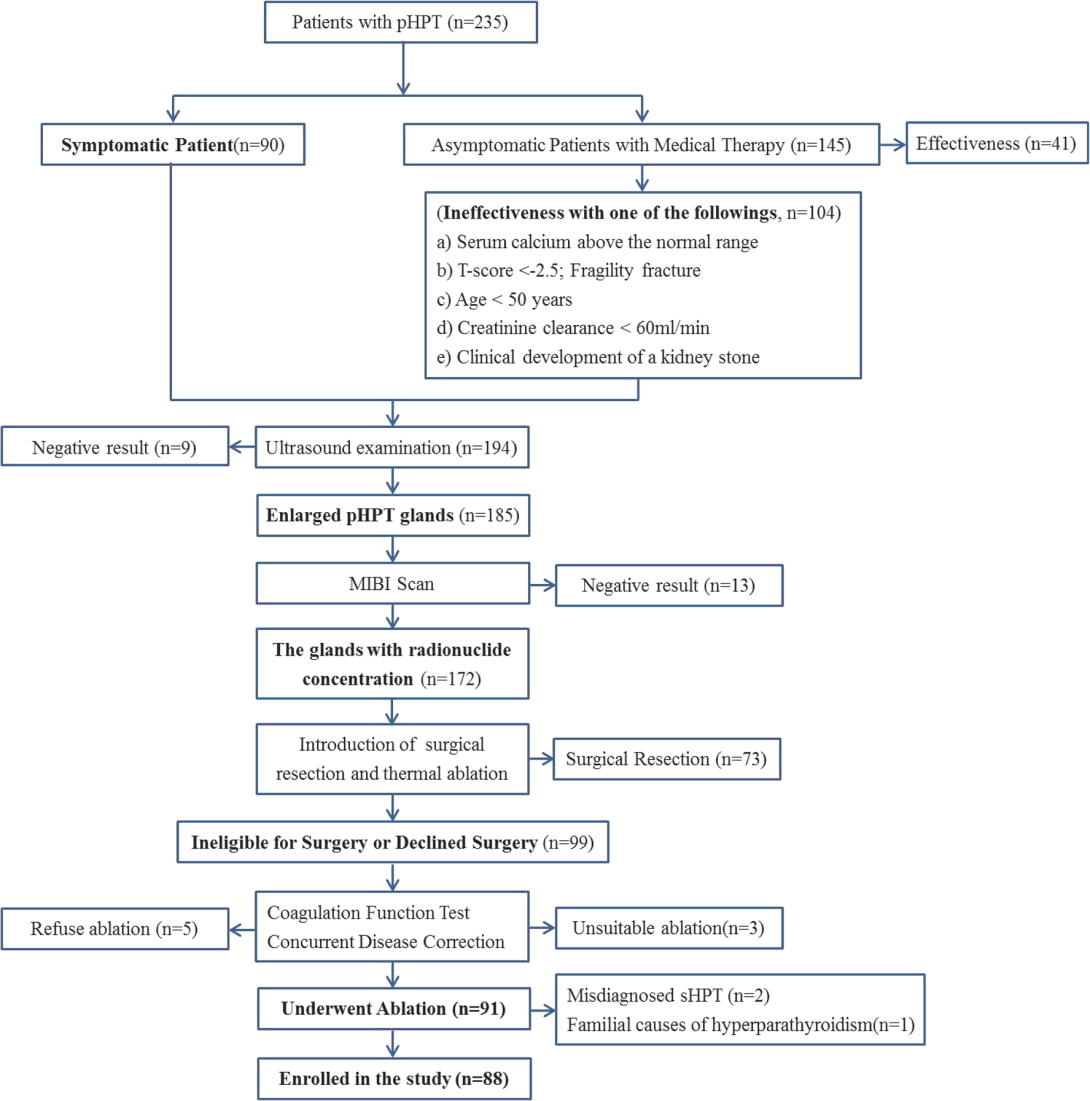
The flowchart of patient selection. pHPT = primary hyperparathyroidism

The exclusion criteria were as follows: (1) patients with severely abnormal coagulation function tests, such as prothrombin time > 18 s, prothrombin activity < 60%, or platelet count < 60 × 10^9^/L; (2) patients with underlying disease, such as cardiac insufficiency or hypertension, refractory to management with medication; and (3) patients with suspected parathyroid carcinoma based on imaging findings and laboratory tests (e.g., enlarged hypoechoic parathyroid glands with unclear margins; lymph node metastasis, markedly elevated iPTH levels and severe hypercalcemia). The flowchart of patient selection is shown in Fig. 1.

According to the guidelines, preoperative parathyroid fine-needle aspiration biopsy is not recommended due to undesirable consequences such as bleeding and needle track implantation [7, 16, 17]. In the present study, the parathyroid nodules of all patients were prelocalized with technetium 99-m-labeled sestamibi single-photon emission computed tomography (99mTc-sestamibi SPECT), routine US, and contrast-enhanced ultrasound (CEUS). Clinical data, biochemical tests, US, CEUS, and 99mTc-sestamibi SPECT were used together for preoperative localization and qualitative diagnosis.

Before MWA, the diagnosis of pHPT on US and CEUS was based on the following criteria: (1) enlarged hypoechoic parathyroid glands with clearly defined margins; (2) no suspicion of lymph node metastasis; and (3) enlarged hypoechoic parathyroid glands showing hyperenhancement in artery phase on CEUS [7, 16, 17].

Information of each patient was obtained, including demographics, clinical parameters, laboratory indices, parathyroid lesion characteristics, and treatment variables (e.g., ablation time, complications, and pre- and post-MWA serum intact PTH [iPTH], calcium, phosphate, alkaline phosphatase [ALP], and 25-OH vitamin D levels). Patients who encountered operative failures were screened out and compared with patients who were successfully cured after MWA. The potential factors associated with operative failures were further analyzed.

### Microwave equipment and ablation technique

The microwave platform (Intelligent Basic Type Microwave Tumor Ablation System, Nanjing ECO Microwave System) contains a microwave generator, a flexible coaxial cable, and a cooled-shaft antenna. The antenna is an internally cooled ablation needle (17-gauge, 10 cm in length, and a 3-mm active tip) covered with polytetrafluoroethylene to prevent adhesion. A LOGIQ E9 US system (GE Healthcare) equipped with a 6–15 MHz linear array transducer was used for US guidance and CEUS.

The patients were placed in a supine position with the neck extended. After the neck was sterilized, 40–60 mL normal saline (NS) was first injected into the area around the parathyroid nodule through an 18-G needle (Hakko Medical Co., Ltd.) to provide hydrodissection. Then, a lidocaine and NS mixture (1:3) was injected near the periparathyroid capsule for local anesthesia. The cooled MWA antenna was inserted freehand into the parathyroid gland under US guidance. During the ablation process, a continuous slow injection of NS was performed by an assistant to provide thermal insulation. A multipoint ablation strategy was adopted [11, 18]. The power was 30 W, and the radiation time was 15–25 s at each ablation point. The therapy was terminated when the hyperechoic zone covered the entire nodule. CEUS (SonoVue; Bracco) was performed 3–5 min later to assess the ablation efficacy. If the ablated nodule was covered by a nonenhanced zone, the ablation was considered complete. If there was nodular enhancement inside the nodule, further ablation was performed immediately. For bilateral nodule ablation, if there were no voice changes and no abnormal vocal cord movements on US after one side was ablated, then MWA would immediately be performed on the contralateral side. If there were any signs of RLN injury, the ablation was stopped, and the second session was suspended until RLN function recovered. At the end of the procedure, the puncture site was compressed for 30 min, and the patient remained under observation for 2 h to monitor for potential complications.

### Follow-up

Follow-up included routine US examination and blood biochemistry. The follow-up times were 2 h, 1 day, 7 days, 1 month, and 3 months after the ablation and every 6 months thereafter. CEUS was only performed if the serum iPTH or calcium levels were elevated or if routine US showed suspicious nodules in the parathyroid region.

### Definition of a cure and operative failure

Technical success was defined as a complete ablation after appropriate treatment according to the protocol. A cure was defined as the reestablishment of normal calcium homeostasis lasting a minimum of 6 months. An operative failure was defined as a failure to normalize serum iPTH and/or calcium levels at 6 months or longer after MWA. Persistent pHPT was defined as a failure to achieve normocalcemia within 6 months. Recurrent pHPT was defined as the recurrence of hypercalcemia and/or elevated iPTH level 6 months after MWA [7, 19].

## Statistical analysis

Statistical analysis was performed using SPSS (version 20.0 for Windows; IBM) and Stata version 15.0 (StataCorp LLC). Continuous data are presented as the mean ± standard deviation, and the median and 25–75% interquartile range (IQR) are used if the data did not fit a normal distribution. Categorical variables were analyzed with the chi-square test, and continuous variables were analyzed with the independent *t*-test or Mann-Whitney *U* test. Differences were considered significant when *p* < 0.05.

## Results

The baseline and treatment parameters are summarized in Table 1. Of the 91 patients reviewed, 2 secondary hyperparathyroidism patients who were misdiagnosed and 1 patient with familial cause of hyperparathyroidism were excluded. Finally, 88 sporadic pHPT patients, consisting of 29 men and 59 women, with 100 parathyroid glands (a single gland in 77 patients, two glands in 10 patients, and three glands in 1 patient) were enrolled. The ages ranged from 18 to 85 years (mean, 56.5 ± 16.8 years). The median maximum diameter of the glands was 1.3 cm (IQR, 0.5–3.4 cm).

**Table 1:** Clinical and treatment characteristics of patients with PHPT

Characteristic	Data
Number of cases	88
Gender (male/female)	29/59
Mean age (years)	56.5 ± 16.8 (18–85)
Symptomatic	40
Nephrolithiasis	16
Ostealgia	16
Fatigue	14
Neurocognitive impairment	2
Asymptomatic	48
25-Hydroxyvitamin D (nmol/L)	31.8 (14.0–81.2)
Pre-MWA iPTH (pg/ml)	143.1 (84.6–563.7)
Pre-MWA calcium (mg/dl)	2.72 ± 0.25 (2.20–3.37)
ALP (U/L)	78 (51–180)
Nodules	100
Normal location	99
Upper pole	28
Lower pole	71
Ectopic location	1
Volume (cm^3^)	0.504 (0.038–6.804)
Maximum diameter (cm)	1.3 (0.5–3.4)
Ablation time (s)	154 (72–368)
Complication	15
Hoarseness	4
Bleeding	3
Transient hypocalcemia	8

### MWA procedure

Complete ablation was achieved in one session in 87 cases and in two sessions in one case. The technical success rate was 100%. The median ablation time was 154 s per nodule (IQR, 72–368 s). The median follow-up duration was 15.9 months (IQR, 6.1–31.5 months). Of the entire cohort, 78 patients had normal levels of serum iPTH and calcium in the follow-up period after MWA, with a cure rate of 88.6% (78/88). The remaining 10 (10/88, 11.4%) patients experienced operative failure. Nine (9/88, 10.2%) patients had persistent pHPT, and 1 (1/88, 1.1%) had recurrent pHPT. The time to recurrence was 9 months after MWA.

### Cause, management, and outcome of patients with operative failure

Of the 10 patients who experienced operative failure, 3 showed increased levels of serum iPTH and calcium, and the other 7 only showed an increase in the serum iPTH level while maintaining a serum calcium level (Table 2).

**Table 2:** The cases of operative failure after MWA

Case	Sex	Age (year)	Nodule diameter (cm)	Pre-MWA			1D post-MWA		Persistent/recurrent PHPT			
				iPTH (pg/mL)	Calcium (mmol/L)	Vitamin D (nmol/L)	iPTH (pg/mL)	Calcium (mmol/L)	Time of detection	iPTH (pg/mL)	Calcium (mmol/L)	Vitamin D (nmol/L)
1	F	73	1.2	139.6	2.45	42.1	31.8	1.96	3 M	121.9	2.56	59.6
2	F	73	1.6	352.0	2.55	24.3	12	2.48	1 M	273.1	2.18	20.8
3	M	75	2.2	230.5	3.01	37.1	15.3	2.82	1 M	88.6	2.86	46.7
4	M	36	0.5	118.8	2.88	42.1	92.2	2.88	1D	92.2	2.88	–
5	F	42	0.4	121.8	2.31	30.7	60.7	2.14	15 M	120.1	2.28	29.4
6	F	61	0.5	103.4	2.43	70.1	96.9	2.51	1D	96.9	2.51	–
7	M	40	1	89.5	2.2	33	119.7	2.19	1D	119.7	2.19	–
8	M	58	0.4	81.8	2.99	46.2	99.7	2.83	1D	99.7	2.83	–
9	M	35	0.9	128.1	2.36	54	98.5	2.39	1D	98.5	2.39	–
10	M	37	0.5	109.4	2.54	37.9	106.3	2.43	1D	106.3	2.43	–

**Fig. 2:**
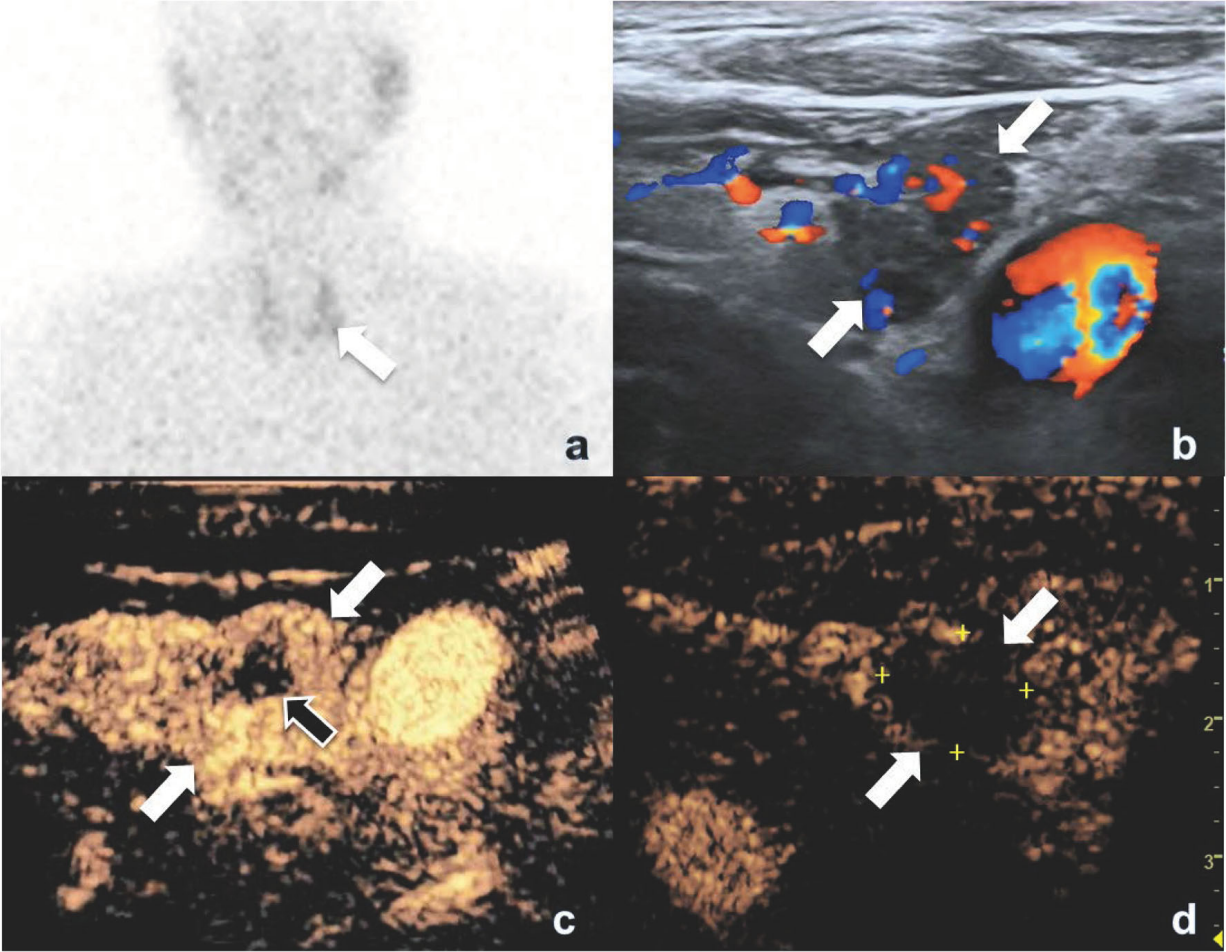
CEUS shows a hyperenhanced residual lesion in a 75-year male patient with pHPT. **a** Three months after the first MWA, there was radioactive concentration in residual lesion (arrow) on MIBI scan. **b** Color Doppler showed abundant blood flow signals around the hypoechoic ablation zone (arrows). **c** There was active area—annular hyperenhancement area—(white arrows) around ablation zone (black arrow) on CEUS. **d** After additional ablation, non-enhancement (arrows) was shown on CEUS. pHPT, primary hyperparathyroidism; CEUS, contrasted-enhanced ultrasound; MWA, microwave ablation; MIBI, technetium 99 m (^99m^Tc) sestamibi

In five of the 10 cases of operative failure, the ablation zone covered the soft tissue close to the pHPT nodule but not the pHPT nodule itself. The maximum diameter of these nodules ranged from 0.4 to 0.5 cm, and the volume ranged from 0.024 to 0.047 cm^3^. For the other two patients with Hashimoto’s thyroiditis, the adjacent lymph nodes rather than the pHPT nodules were ablated. The lymph nodes were near the parathyroid nodules (maximum diameter, 0.9–1.0 cm; volume, 0.196–0.203 cm^3^). In the other 3 cases, incomplete ablation was found by elevated serum iPTH levels 1–3 months after MWA. The residual lesions showed hyperenhancement on CEUS (Fig. 2).

Of these 10 patients, three with hypercalcemia underwent successful surgical resection (2 patients) or a second ablation (1 patient), respectively. Three patients with residual lesions received additional ablation and were cured. The other 4 patients with very small nodules and only elevated serum iPTH levels received vitamin D, calcitriol supplementation, and regular follow-up. The causes, management, and outcomes of patients with operative failures after MWA are outlined in Table 3.

**Table 3:** The causes of operative failure after MWA

Cause of operative failure	Number of case (*n*)	Management	Outcome
Failed to ablate target lesion	7	–	–
-Too small nodules (diameter ≤ 0.5 cm)	5	Surgery (1), ablation (1), medical treatment (3)	Cure
-Confused with lymph nodes	2	Surgery (1), medical treatment (1)	Cure
Incomplete ablation	3	Second ablation	Cure

### Risk factors for operative failure

There were significant differences in the pre-MWA iPTH level (*p* = 0.016), serum calcium level (*p* = 0.03), lesion diameter (*p* = 0.000), and lesion volume (*p* = 0.000) between the cured patients and the patients with operative failure due to missed or false punctures (Table 4). Serum calcium (*r* = 0.241, *p* = 0.027) and iPTH (*r* = 0.560, *p* = 0.000) values were positively correlated with the maximum diameter of the parathyroid nodule. According to the ROC analysis, a maximum diameter less than 0.6 cm or a volume smaller than 0.208 cm^3^ resulted in the highest Youden index values for operative failure. Of the nine patients with lesions with a maximum diameter less than 0.6 cm, 77.8% (7/9) of the patients encountered operative failure.

**Table 4:** Comparison of relevant clinical parameters between the cured cases and the cases of operative failure due to missing and false puncture

Variables	Operative failure (7)	Cure (77)	*p*
Female (*n*)	3	53	0.215
Age (years)	44.1 ± 10.8	56.9 ± 17.0	0.055
Pre-MWA iPTH (pg/ml)	109.4 (81.8–121.8)	151.8 (86.3–579.4)	0.016
Serum calcium (mmol/L)	2.53 ± 0.30	2.74 ± 0.24	0.030
Serum phosphorus (mmol/L)	0.95 ± 0.18	0.84 ± 0.19	0.160
ALP (U/L) Regenerative growth	68 (54–85)	78 (50–195)	0.124
25(OH)D3(nmol/L)	42.1 (30.7–54)	32.8 (13–83.5)	0.054
GFR (mL/min)	95.9 (83.1–114.2)	97.6 (41.4–128.5)	0.492
CCR (umol/L)	77 (45.5–91.8)	60.1 (43.1–108.3)	0.345
Ur (mmol/L)	4.5 (2.2–7.4)	4.3 (3.3–5.9)	0.654
Max diameter (cm)	0.5 (0.4–0.9)	1.3 (0.6–3.6)	< 0.001
Volume (ml)	0.042 (0.024–0.196)	0.580 (0.074–8.709)	< 0.001

*iPTH*, intact parathyroid hormone; *ALP*, alkaline phosphatase; *MWA*, microwave ablation; *GFR*, glomerular filtration rate; *CCR*, creatinine clearance rate; *Ur*, urea

In the three patients that underwent incomplete ablation, the univariate analysis results showed that there were no parameters associated with operative failure.

### Complications

As a major complication, voice change occurred in 4 patients (4.5%), but all improved within 2–3 months. As a minor complication, hematoma occurred in 3 (3.4%) patients, and all hematoma was treated successfully with pressure and thrombin injections (Hemocoagulase Bothrops Atrox for Injection, Nuokang Pharmaceutical Company). Mild postoperative transient hypocalcemia accompanied by perioral and limb numbness occurred in 8 patients (9.1%). Their symptoms were gradually relieved by the appropriate use of calcitriol and supplemental calcium.

## Discussion

In recent years, US-guided thermal ablation for pHPT has been demonstrated to be a promising option because of its minimal invasiveness, safety, promising efficacy, wide indications, and rapid postoperative recovery [8–12]. However, the cure rate of MWA is approximately 82.1–89.4%, and still there are some patients have failed procedures [9–11]. Therefore, it is essential to summarize the cases of operative failure after MWA, analyze the possible reasons to prevent these situations, and increase the cure rate.

**Fig. 3:**
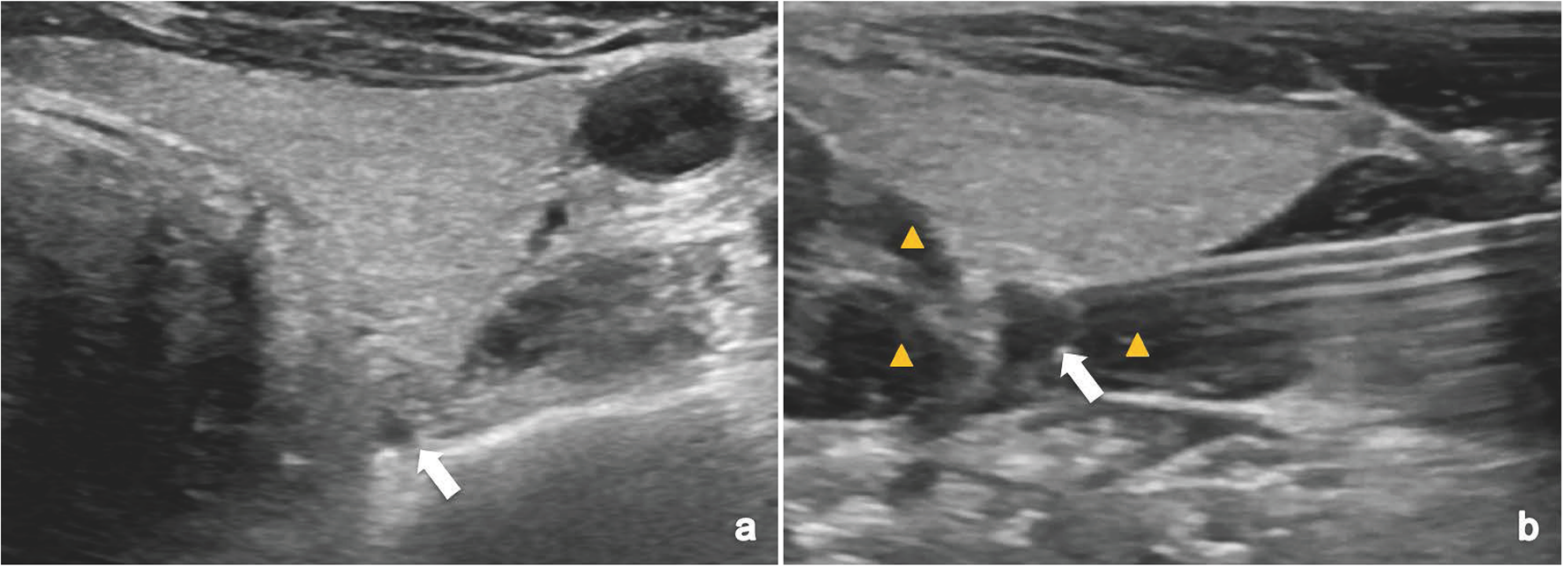
Nodular changes in the surrounding tissues during hydrodissection. **a** Routine US showed a hypoechoic pHPT nodule with maximum diameter of 0.4 cm in a 58-year male patient. **b** During hydrodissection, the liquid (yellow arrow) diffuse unevenly and cause nodular changes in the surrounding tissues. Both the liquid and the pHPT nodule (arrow) were hypoechoic. pHPT, primary hyperparathyroidism

This retrospective study reviewed 88 patients who underwent MWA for sporadic pHPT. Up to now, the present study included the largest number of pHPT patients who have undergone MWA [9, 10, 12]. The results demonstrated operative failure in 11.4% of patients. According to the statistical results, when the maximum diameter of the parathyroid nodules was less than 0.6 cm, the possibility of operative failure was obviously higher, at 77.8%. According to our experience, during hydrodissection, the liquid can diffuse unevenly and cause nodular changes in the surrounding tissues. As a result, the echogenicity of the liquid was similar to that of the parathyroid nodules—both were hypoechoic, which easily misguided the operator and led to false targets (Fig. 3). In fact, some small parathyroid nodules might be missed even during open surgery [14]. In cases of missed puncture, even if the thermal ablation zone was close to the nodule, it was difficult for the heat energy to reach the pHPT nodule due to the barrier of the nodule capsule and the heat sink caused by the isolation fluid, thereby causing incomplete ablation. Therefore, several strategies could be employed to prevent false puncture: (i) before injecting the isolation solution, the PTC needle could be inserted into the nodule to anchor the nodule; (ii) the ablation antenna could be directly inserted into the nodule first, before establishing hydrodissection. In other words, prelocalization should be adopted to avoid losing the target nodules and prevent operative failure for patients with small lesions.

For patients with lymphocytic thyroiditis, the perithyroidal central compartment lymph nodes can commonly be misdiagnosed as parathyroid nodules [19]. Some parathyroid nodules were similar to the lymph nodes in size and echogenicity, making it difficult to distinguish between the two and sometimes resulting in false puncture during ablation. In these cases, CEUS could be helpful. According to our experience, the lymph nodes in patients with Hashimoto’s thyroiditis were usually hypoenhanced in the arterial phase on CEUS, while the pHPT nodules due to hypermetabolism were generally hyperenhanced.

**Fig. 4:**
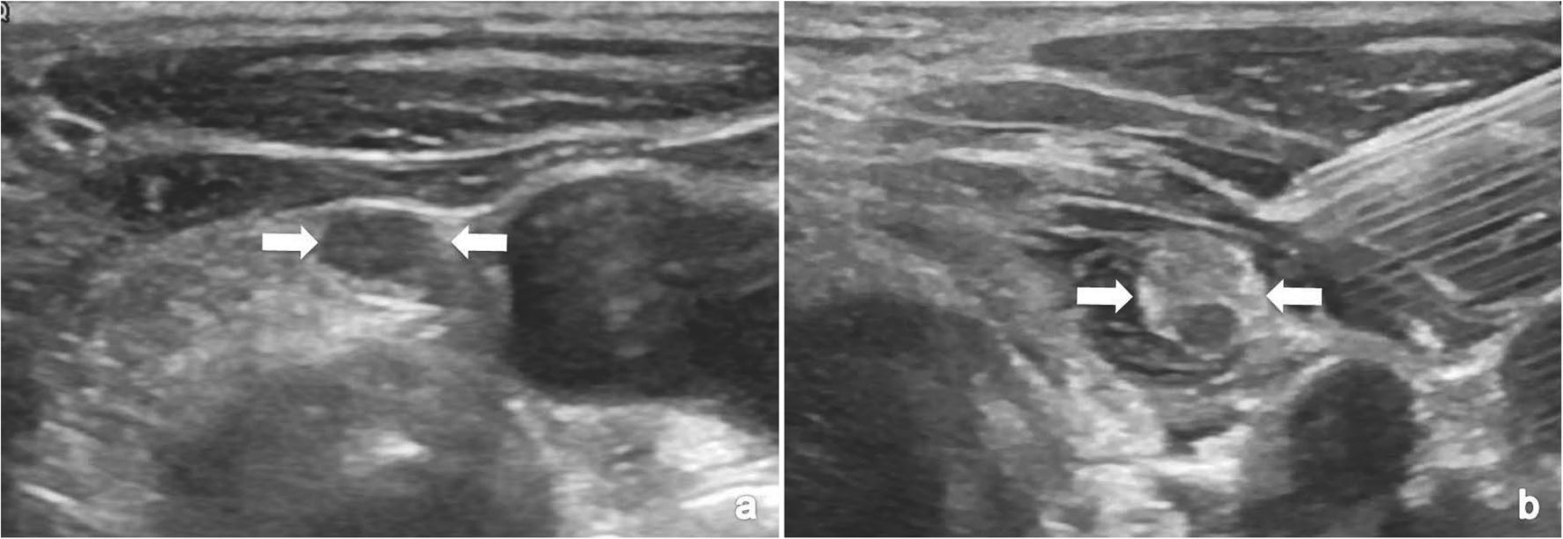
The hypoechoic hyperplastic parathyroid nodule and hyperechoic normal parathyroid tissue could be clearly visualized after establishment of hydrodissection. **a** Routine US showed a hypoechoic pHPT nodule (white arrows) in a 45-year female patients. **b** After the injection of isolation solution, the hypoechoic hyperplasic parathyroid nodule (white arrows) and hyperechoic normal parathyroid tissue were clearly displayed on US. pHPT, primary hyperparathyroidism; US, ultrasound

Partial remission of hyperparathyroidism caused by incomplete ablation had also been reported in previous studies [10, 20]. Based on the three patients with operative failure due to incomplete ablation in the present study, our recommendations are summarized as follows: (i) a complete ablation should contain both hypoechoic hyperplastic nodules and normal parathyroid tissue, which often appear hyperechoic on US, especially after the injection of the isolation solution (Fig. 4); and (ii) intraoperative iPTH monitoring is one of many adjuncts to achieve complete ablation according to the surgical procedure [21].

Transient hoarseness caused by impaired mobility of the ipsilateral vocal cord was reported in previous studies on thermal ablation of pHPT. The rate was varying from 6 to 38% [11, 12, 22]. In the present study, hoarseness occurred in 4 patients (4.5 %), but all patients’ voice improved within 2–3 months. The rate was higher than that reported in the thermal ablation of thyroid nodules (1.5%) and in parathyroidectomies (2.4%) [23, 24]. The reason might be that some parathyroid nodules were too close to the tracheoesophageal groove, where the recurrent laryngeal nerve (RLN) was located; and the nerves were very sensitive to thermal stimulation. Therefore, minimizing heat exposure to the RLN is extremely critical during ablation. There are several strategies that could be employed to protect the nerve. First, effective hydrodissection technology can effectively reduce thermal damage to the RLN. Second, accurate puncture and ablation monitored by US could help protect RLN against thermal injury. Third, short time and repeated radiation with low power is helpful in preventing thermal injuries to the surrounding critical structures.

There are still a few limitations in the present study. First, no pathological results were obtained because biopsy was not recommended according to the guidelines. The number of operative failures was relatively small, and future studies with large sample sizes might help to provide more definitive results. Third, this is a retrospective study, and there may be selection bias. Prospective studies are needed.

In conclusion, MWA is an effective treatment method. However, operative failure is still inevitable in 11.4% patients. The small size (maximum diameter < 0.6 cm) of pHPT nodule was one of the key factors leading to operative failure. Prelocalization and mastery of the details of the ablation technique could help avoid operative failure and improve the cure rate by increasing the rate of complete ablation.

## Abbreviations

CEUS: Contrast-enhanced ultrasound
MWA: Microwave ablation
pHPT: Primary hyperparathyroidism
PTH: Parathyroid hormone
US: Ultrasound

## Declarations

### Guarantor

The scientific guarantor of this publication is corresponding author Yu Ming-an.

### Statistics and biometry

Data analysis has been finished by Ying Wei and no complex statistical methods were necessary for this paper.

### Methodology

• retrospective/observational study
